# Centromere function in asymmetric cell division in *Drosophila* female and male germline stem cells

**DOI:** 10.1098/rsob.210107

**Published:** 2021-11-03

**Authors:** Antje M. Kochendoerfer, Federica Modafferi, Elaine M. Dunleavy

**Affiliations:** Centre for Chromosome Biology, Biomedical Sciences, National University of Ireland Galway, Galway H91 TK33, Ireland

**Keywords:** centromere, CENP-A, germline stem cell, asymmetric cell division, epigenetics, *Drosophila*

## Abstract

The centromere is the constricted chromosomal region required for the correct separation of the genetic material at cell division. The kinetochore protein complex assembles at the centromere and captures microtubules emanating from the centrosome to orchestrate chromosome segregation in mitosis and meiosis. Asymmetric cell division (ACD) is a special type of mitosis that generates two daughter cells with different fates. Epigenetic mechanisms operating at the centromere have been proposed to contribute to ACD. Recent studies have shown that an asymmetric distribution of CENP-A—the centromere-specific histone H3 variant—between sister chromatids can bias chromosome segregation in ACD. In stem cells, this leads to non-random sister chromatid segregation, which can affect cell fate. These findings support the ‘silent sister' hypothesis, according to which the mechanisms of ACD are epigenetically regulated through centromeres. Here, we review the recent data implicating centromeres in ACDs and cell fate in *Drosophila melanogaster* female and male germline stem cells.

## Graphical abstract

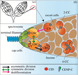

## 1. Introduction

Stem cells are unspecialized cells that are essential for the generation of tissues of all metazoans during embryogenesis and throughout adult-life. Stem cells often undergo asymmetric cell division (ACD), during which each stem cell generates two daughter cells with distinct fates [1]. ACD is critical to preserve stem cell populations in multicellular organisms and to balance the production of differentiating cells. Disruption of this balance can lead to tumorigenesis triggered by stem cell over-production or to tissue degeneration caused by stem cell depletion [2]. To prevent such outcomes, homeostatic conditions ensure a certain net number of each cell type such that cell loss is compensated by the production of new cells. In addition to ACD, stem cells can also undergo symmetric divisions to give rise to two identical daughter stem cells [3]. In some instances, differentiated daughter cells can dedifferentiate in order to replenish lost stem cells [4].

Epigenetic mechanisms were previously proposed to play an important role in defining distinct cell fates during ACD [5,6]. The ‘silent sister' hypothesis (SSH) stated that in stem cells, ACD occurs through the marking and recognition of epigenetically distinct sister chromatids [5]. According to this hypothesis, two sister chromatids have different epigenetic marks, which can lead to a different gene expression pattern in the stem and daughter cells. In stem cells, these marks are responsible for the expression of stemness genes and the silencing of differentiation genes; in the differentiated daughter cell, differentiation genes are expressed while stemness genes are ‘silent’. In support of this hypothesis, evidence for epigenetically distinct sister chromatids has emerged in *Drosophila melanogaster* germline stem cells (GSCs) [7,8] and in mouse embryonic stem cells [9]. More specifically, the SSH proposed that epigenetic differences at centromeres, the primary constriction on chromosomes where spindle microtubules attach [10], lead to non-random sister chromatid segregation. As a result, recent efforts have focused on studying potential epigenetic differences between sister centromeres in stem cells.

The *Drosophila melanogaster* GSC niche is relatively simple and well defined, and offers an ideal model system in which to test the SSH. *Drosophila* centromeres are composed of CID (Centromere Identifier, the centromere-specific histone H3 variant and CENP-A homologue), its centromere-specific chaperone and assembly factor CAL1 (Chromosome Alignment Defect 1) and CENP-C (Centromere Protein C) that links it to the kinetochore [11–13]. Correct interplay between these proteins is essential for proper centromere function and CID assembly at centromeres each cell cycle [14,15]. Recent studies in *Drosophila* GSCs have shown that during ACD sister chromatids harbour asymmetric levels of CID, with more CID on the chromosomes that end up in the future stem cell as opposed to those that end up in the daughter cell [16,17]. Enhanced microtubule capture by these ‘stronger' centromeres can potentially bias sister chromatid segregation in ACD. These centromere-based studies in *Drosophila* have led to the proposal of a ‘mitotic drive' model in stem cells [18,19], inspired by previous ‘meiotic drive' models that implicated centromere strength in biased homologous chromosome segregation [20–22]. Additional functional studies involving the over-expression and knockdown of CID, CAL1 and CENP-C have demonstrated important roles not only in asymmetric centromere assembly in GSCs but also in cell fate [16,17,23]. We discuss these recent data which provide support for the SSH and mitotic drive in both female and male *Drosophila* GSCs.

## 2. *Drosophila* female germline stem and daughter cells

**Figure 1.**
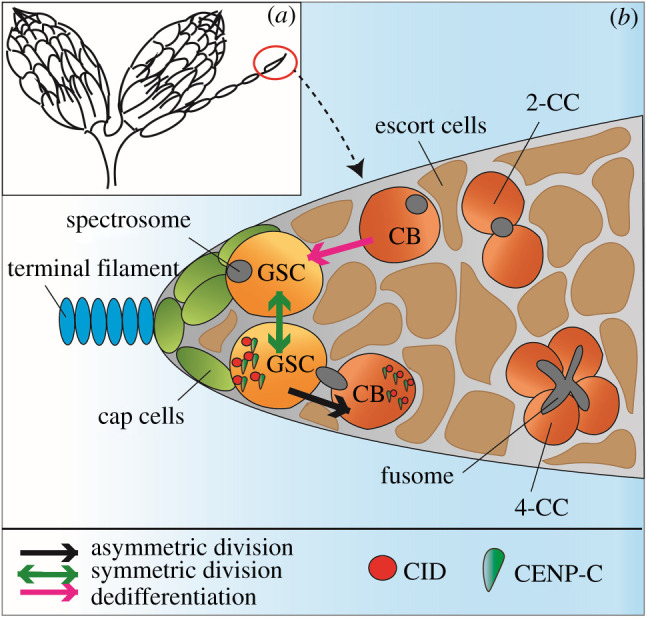
Female *Drosophila* ovaries and germline stem cell (GSC) niche. (*a*) Cartoon of the overall structure of *Drosophila* ovaries. Each of the two ovaries consists of 16 ovarioles. The germarium is located at the anterior end of the ovariole (red circle). Ovarioles consist of egg chambers connected by bridges. (*b*) The germarium hosts 2–3 GSCs interacting with cap cells (light green) attached to the terminal filament (blue) in the stem cell niche. GSCs and daughter cystoblasts (CBs) are surrounded by escort cells (brown). GSCs can be identified by the round spectrosome organelle (grey), closely attached to the cap cells. GSCs can divide asymmetrically in order to generate another GSC and a differentiating CB (black arrow). CBs divide by mitosis to give 2-cell cysts (2-CC). In total, the CB undergoes four mitotic divisions to produce a 16-cell cyst that completes meiosis to generate an oocyte and 15 nurse cells (not shown). The branched spectrosome, called the fusome (grey) is present at the 4-cell cyst stage (4-CC) and in following mitotic divisions. GSCs and CBs enter synchronously into S-phase and can be identified by a bridge-shaped spectrosome. At S-phase, centromere proteins CID (red) and CENP-C (green) are asymmetrically distributed, with more present in the GSC compared to the CB. GSCs can divide symmetrically to produce two GSCs (green arrow). Differentiated CBs and CCs up until the 8-cell cyst stage (not shown) can potentially dedifferentiate to replenish lost GSCs (pink arrow).

The *Drosophila* adult female gonad comprises a pair of ovaries, each consisting of 16 ovarioles [24]. At the apical end of each ovariole lies the germarium, containing the stem cell niche at its anterior tip (figure 1*a*). The stem cell niche is made of 2 to 3 GSCs and supporting cap and terminal filament cells [25,26]. Female GSCs undergo ACD to generate two daughter cells: a GSC to renew the stem cell pool and a differentiating daughter cell, called the cystoblast (CB), both supported by escort cells. GSCs are morphologically distinguishable by their attachment to the cap cells and the presence of an anteriorly localized round spectrin-rich organelle, called the spectrosome [27]. The spectrosome is associated with one pole of the mitotic spindle and orients ACD [28,29]. Proceeding away from the niche, the CB that also harbours a round spectrosome, undergoes four mitotic divisions without complete cytokinesis to generate 2-, 4-, 8- and 16-cell cysts of cystocytes (CCs). 4-, 8- and 16-cell cysts can be identified by the elongated morphology of the spectrosome, now called a fusome that interconnects CCs (figure 1*b*). Among the 16 cells, one will complete meiosis to become the oocyte whereas the other 15 will become nurse cells, which synthesize proteins and mRNAs, and transfer them to the oocyte [24]. At the distal end of the ovariole lies the egg chamber containing the mature oocyte ready for fertilization.

GSCs and CBs acquire different fates through signalling molecules, such that GSCs can proliferate and renew the stem cell pool, whereas CBs will start to differentiate and will eventually enter into meiosis. During embryo development, *nanos (nos)* mRNA is initially required, when its expression inhibits division of the germ cell precursor pole cells, allowing these cells to migrate to the forming gonad [30]. Later, *nos* expression is crucial for GSC maintenance in the niche [30]. In the cap cells, Janus Kinase and Signal Transducer and Activator of Transcription (JAK-STAT) signalling activates the bone morphogenetic protein (BMP) ligands *Decapentaplegic (Dpp*) and *Glass bottom boat (Gbb*). BMP activation promotes GSC self-renewal by repressing differentiation-promoting genes through phosphorylation of *Mad* (*Mothers against Dpp, pMad*) [31–34]. An important factor for GSC differentiation is *bag of marbles (bam*), which is repressed by *pMad* and is expressed in 4- and 8-cell cysts [31,34–36]. In the case of a reduction in the number of female GSCs, the pool can be replenished by symmetric divisions, meaning a GSC will divide to generate two new GSCs [37] or by dedifferentiation of 4- and 8-cell cysts [38] (figure 1*b*).

## 3. *Drosophila* male germline stem and daughter cells

**Figure 2.**
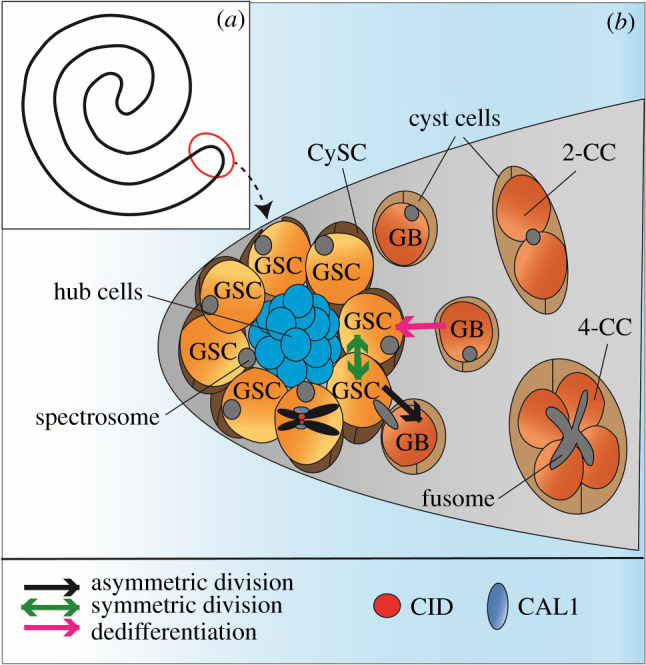
Male *Drosophila* testis and germline stem cell (GSC) niche. (*a*) Cartoon of one of the two spiral-shaped *Drosophila* adult testes. The germline stem cell niche is located at the apical tip of the testes (red circle). (*b*) In males, 6–15 GSCs surround 10–15 non-dividing hub cells (blue). GSCs are surrounded by two cyst stem cells (CySCs) (dark brown) and daughter cells, called gonialblasts (GBs) are enclosed by two cyst cells (light brown). The round spectrosome organelle (grey) is characteristic for GSCs. GSCs can divide asymmetrically in order to generate another GSC and a differentiating GB (black arrow). GBs divide by mitosis to give 2-cell cysts (2-CC). In total, the GB undergoes four mitotic divisions to produce a 16-cell cyst that completes meiosis to generate 64 haploid spermatids (not shown). The branched spectrosome, called the fusome (grey) is present at the 4-cell cyst stage (4-CC) and elongates further in following mitotic divisions. GSCs at prometaphase/metaphase, illustrated by a single pair of sister chromatids in black, display an asymmetric distribution of CID (red) and CAL1 (blue) between sister centromeres, with more present on the sister chromatid to be inherited by the GSC. GSCs can divide symmetrically to produce two GSCs although this is a rare event (green arrow). Differentiated GBs and CCs mostly up until the 2-CC stage can potentially dedifferentiate to replenish lost GSCs (pink arrow).

The *Drosophila* adult male gonad comprises two spiral-shaped testes, each with the GSC niche located at its apical end (figure 2*a*). Each *Drosophila* testis hosts 6–15 GSCs surrounding 10–15 non-dividing stromal cells, which form the hub region, organized by cell-cell adhesion molecules such as integrins and cadherins [4,25,26]. The number of GSCs can vary between different fly strains [4]. In the niche, male GSCs undergo ACD to generate another GSC remaining at the hub and a gonialblast (GB) that relocates away from it. Comparable to female GSCs and CBs, male GSCs and GBs are characterized by a round spectrosome organelle (figure 2*b*). GSCs are encased between two cyst stem cells (CySCs), while GBs are surrounded by two non-dividing somatic cyst cells [4]. Again comparable to CBs, GBs enter into four rounds of mitosis to develop into a cyst of 16 spermatogonial cells, which enter into G2 phase and meiosis I to generate 16 primary spermatocytes. After the first meiotic division, a cyst of 32 secondary spermatocytes enters into the second meiotic division generating a cyst of 64 round, haploid spermatids [39]. Towards the end of spermatogenesis, spermatids individualize by generating their own plasma membranes, and flagella elongate to eventually form mature adult spermatozoa. Highly condensed needle-like spermatozoa are stored in the seminal vesicle, a tubular structure located at the distal end of the testes [40].

Male GSCs receive signalling through JAK-STAT activation, which is stimulated by the cytokine-like ligand *Unpaired (Upd)* secreted from hub cells [41–43]. Hub cells and CySCs also secrete BMP ligands *Dpp* and *Gbb*. Binding of *Dpp* and *Gbb* to receptors in adjacent GSCs leads to the activation of BMP signalling where *pMad* prevents transcription of *bam* [41,44–46]. Repression of *bam* was also reported in GBs [45]. To ensure ACD, the mitotic spindle of the GSC is positioned perpendicular to the hub region, the orientation of which is set by the centrosome [47,48]. This orientation results in a reduction in JAK-STAT signalling in the GB that subsequently differentiates [25,42,43] (figure 2*b*). Inheritance of the mother centrosome by the GSC is critical for this mechanism [49]. Symmetric division also occurs in males, where one GSC divides into two identical GSCs to replace lost stem cells [4], although this is a rare event. Extended live imaging of the male GSC niche has shown that GSC-GB pairs can swivel back to contact the hub and undergo symmetric division [50]. With a loss of GSCs caused by manipulating JAK-STAT signalling or over-expressing *bam*, differentiated cells up until the 8-cell cyst stage can dedifferentiate to replenish the GSC pool, although it mostly happens from the 2-cell cyst stage [50]. For this to occur, daughter cells revert back to the hub and can potentially regain stem cell fate [51]. In spermatocytes, dedifferentiation is not possible as this cell type is terminally differentiated [51] (figure 2*b*).

## 4. Epigenetic mechanisms contribute to stem cell identity: the ‘silent sister' hypothesis and ‘mitotic drive' via centromeres

**Figure 3.**
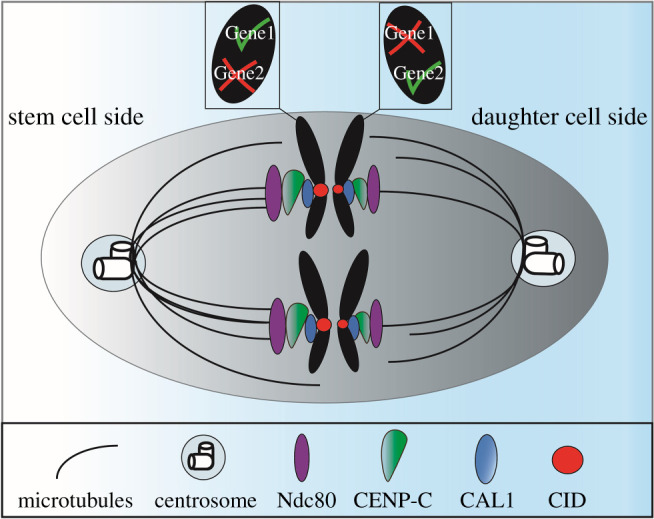
The silent sister hypothesis and mitotic drive. Epigenetic differences at centromeres can influence stem cell identity. The selective attachment of microtubules can lead to non-random segregation of sister chromatids in ACD in GSCs. The future stem cell side (left) shows a higher amount of centromeric proteins CID (red), CAL1 (blue) and CENP-C (green), as well as the outer kinetochore protein Ndc80 (purple), than the future daughter cell side (right). This generates stronger kinetochores interacting with more microtubules that biases sister chromatid segregation. Different epigenetic marks at specific genes can affect gene expression. The future stem cell will inherit the sister chromatid in which differentiating genes are silenced (gene 2) and genes important for stemness (gene 1) are activated. The future daughter cell will inherit the sister chromatid with genes active for differentiation (gene 2) and will undergo mitosis and ultimately meiosis.

Previously, the SSH proposed that ACD of stem cells is facilitated by marking epigenetically distinct sister chromatids [5] (figure 3). Epigenetic mechanisms have been shown to contribute to ACD at the level of histone inheritance. Using a dual colour labelling technique that can distinguish pre-existing from newly synthesized histones, it was shown that canonical histones H3 and H4 are asymmetrically distributed in dividing male GSCs [7,52]. Globally, newly synthesized histone H3 or H4 is enriched in the GB and pre-existing histone H3 or H4 is enriched in the GSC. More recently, the preferential retention of pre-existing histones H3 and H4 in female GSCs was also observed, but only at specific loci [53]. DNA oligopaint experiments showed that pre-existing histones are enriched at genes associated with stemness in GSCs and newly synthesized histones are enriched at genes that promote differentiation in GBs. Therefore, whether global or local, the specific retention of ‘old’ histones in stem cells and ‘new' histones in differentiating cells seems to be a feature of both male and female GSCs. A similar phenomenon was reported for CID in the *Drosophila* midgut. Del Arco *et al.* showed that intestinal stem cells (ISCs) harbour an asymmetric distribution of ‘old' and ‘new' CID between ISCs and differentiating enteroblasts (EBs) [54]. In addition to the differential distribution of old and new histones, an overall asymmetry in the total amount of CID present in stem and daughter cells has been observed. Our recent study in *Drosophila* female GSCs demonstrated that at metaphase, sister chromatids which end up in the stem cell contain 20% more CID at centromeres [17]. Also at metaphase, more microtubules were observed on the stem cell side. Consistent with findings in females, an asymmetric distribution of CID was detected in *Drosophila* male GSCs at pro-metaphase with an approximate 50% enrichment of CID on sister chromatids that end up in the stem cell versus the daughter cell [16]. Furthermore, an asymmetric distribution for CENP-C was also detected in female GSCs [17,23], as well as the outer kinetochore protein Ndc80 in males [16] (figure 3). Taken together, these studies propose that enhanced centromere, kinetochore and microtubule strength mediate non-random sister chromatid segregation in GSCs [16,17]. In males, the differential timing of nuclear envelope breakdown, with the GSC-side nuclear envelope breaking down earlier in G2-phase, facilitates a bias in microtubule attachment [16]. Accordingly the ‘mitotic drive' model, posing a biased inheritance of cell components during mitosis, applies to these stem cell populations [18,19] (figure 3). Interestingly, diverse organelles, for example the centrosome [49] and midbody ring [55], show an asymmetric distribution in GSCs. In both males and females, GSCs inherit the centrosome with the highest MTOC activity [49,55]. Interestingly, in female GSCs the centrosome with the highest MTOC activity is the daughter centrosome [49,55]. Therefore, centromere and microtubule bias towards the GSC side in both females and males [16,17] does not seem to correlate with inheritance of the mother centrosome. It appears that GSC centromeres are stronger independently of mother or daughter centrosome retention. For this reason we suggest that centromere and kinetochore strength can be a primary driver of asymmetric spindle assembly in *Drosophila* GSCs. In addition, phosphorylation of histone H3 at threonine 3 (H3T3P) was shown to play a role in biasing non-random sister chromatid segregation [8]. The sister chromatid which is inherited by the stem cell is more heavily phosphorylated at this residue, than the sister chromatid which is segregated towards the daughter cell side [8]. Intriguingly, depletion of HASPIN in female GSCs, the kinase responsible for the H3T3P mark [56], leads to a 65% increase in CID level at centromeres, as well as a loss in CID asymmetry [17]. Therefore, H3T3P/HASPIN specifically impacts on centromere assembly and asymmetry, providing an additional mechanism by which chromosome segregation might be skewed in GSCs. Together, these findings support a role for epigenetic mechanisms at centromeres in controlling ACD in the *Drosophila melanogaster* germline both in females and males. Based on these recent data, following on from the SSH, the mitotic drive model places stronger centromere and microtubule attachments as key features that can bias sister chromatid segregation in ACD.

## 5. Centromere assembly in *Drosophila* GSCs: timing and mechanism

**Figure 4.**
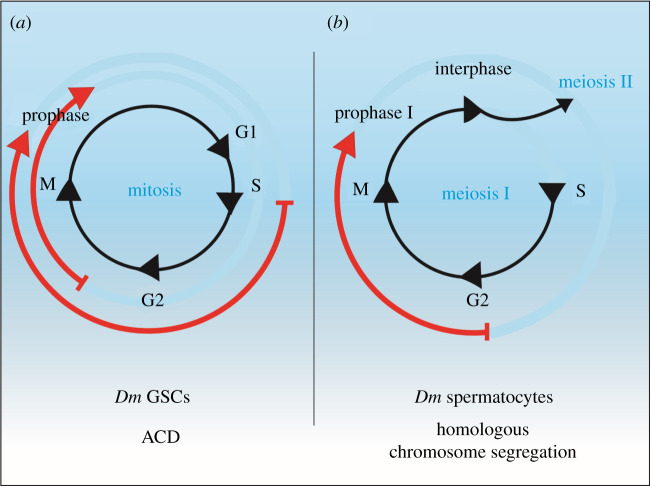
Comparison of CID assembly time points in *Drosophila* GSCs and spermatocytes. (*a*) In *Drosophila* GSCs, which undergo ACD, CID is assembled between S-phase and G2-phase up to prophase of mitosis (M) in females (outer red arrow) and from mid/late G2-phase up to prophase or early mitosis in males (inner red arrow). A short G1-phase is characteristic of these cells. (*b*) In *Drosophila* spermatocytes, which undergo homologous chromosome segregation in meiosis I, CID is assembled between G2-phase up to prophase I (red arrow).

CENP-A is a histone H3 variant that must be assembled each cell cycle to replenish its level following each division and to maintain a functional centromere [57]. Pre-existing CENP-A is diluted during S-phase as it is redistributed equally between sister chromatids [58]. Unique from canonical histones (H2A, H2B, H3, H4) that are assembled at the DNA replication fork, newly synthesized CENP-A assembles in a replication-independent manner [59]. In human HeLa cells in culture, CENP-A is assembled at late telophase/early G1-phase [58]. In *Drosophila*, CID assembly was first characterized in embryonic cells, occurring at anaphase [60]. Later studies in cultured mitotic cells showed that CID is assembled at metaphase or early G1-phase [61–63]. Recently, the timing of CID assembly in stem cells has been elucidated. Female *Drosophila* GSCs assemble CID between DNA replication and prophase [17]. Investigations in male GSCs reported a similar timing for CID assembly, characterized from mid/late G2-phase up to prophase or early mitosis [16] (figure 4*a*). Potentially, GSCs might use this assembly time point mechanistically to distinguish epigenetically distinct sister centromeres. For example, centromere assembly before chromosome segregation, could allow the GSC to first establish CID asymmetry before loading the other mitotic components. Interestingly, this timing is somewhat comparable to that of CID assembly during meiosis, occurring at prophase I [63,64] (figure 4*b*). In this instance, CID assembly might be required to modify the centromere/kinetochore configuration in preparation for homologous chromosome segregation. However, whether sister centromere asymmetry in GSCs already occurs during centromere replication in S-phase and pre-exists centromere assembly or is actively established in G2-phase remains unknown. It is possible that this specific time window might also serve in other stem cell types of metazoans to initiate the mechanism of ACD.

In humans, new CENP-A assembly involves the Mis18 complex and HJURP (CAL1 equivalent) [65–69]. Interestingly there is no known Mis18 complex homologue in *Drosophila* [70]. Indeed, the 16-component constitutive centromeric associated network (CCAN) isolated in human cells [71] is almost completely missing in *Drosophila*, with the exception of CENP-C. Therefore, *Drosophila* offers a simplified centromere, with its assembly and function reliant on only three proteins (CID, CENP-C and CAL1). Recent data generated from cultured cells, as well as structural studies, have elucidated a self-propagating loop of CID maintenance and inheritance [15,72]. According to this model, the CAL1 C-terminus recognizes CENP-C bound to pre-existing CID nucleosomes and CAL1 recruits one new CID-H4 dimer through its N-terminus. Self-association of CAL1 is required for new CID deposition at centromeres. Following, CENP-C dimers associate with newly assembled CID nucleosomes and interact with CAL1 via their C-termini. Finally, CAL1 recruits and loads new CENP-C to maintain this epigenetic loop. The CENP-C N-terminus connects the centromere with the kinetochore and becomes the platform for its assembly. It is not known if this mode of assembly applies to GSCs, or indeed any stem cell population. It would therefore be interesting to investigate centromere assembly timing and Mis18 complex/HJURP functions in human stem cell populations.

## 6. Evidence supporting centromere function in GSC fate

Models of ‘mitotic drive’ propose that centromere asymmetry directs ACD [18,19] (figure 3). But if and how this impacts on cell fate remains enigmatic. Functional studies, again in *Drosophila* female and male GSCs, have recently attempted to shed light on this [16,17,23]. In female GSCs, CID and CAL1 knockdown resulted in germaria with very few or no germ cells [17]. This result is perhaps not surprising, given the essential function of both genes in cell division. Partial depletion of CENP-C (an approximate 60% reduction of CENP-C in GSCs) was however possible in females, which allowed phenotypic analysis [23]. In this case, a reduction in CENP-C level enhanced CID asymmetry such that even more CID is retained by the GSC. In parallel, a disruption to the balance of stem and daughter cells in the niche was observed. Using pMad to mark stem cells and Sex-Lethal (SXL) to mark the GSC to daughter cell transition, the calculated SXL/pMad ratio indicated more GSCs relative to differentiating cells. This result suggested a shift towards GSC self-renewal upon disrupted centromere asymmetry. Interestingly, CENP-C knockdown after 10 days resulted in germaria that had lost the GSC pool, implicating CENP-C in GSC long-term maintenance [23]. Therefore, it appears that a reduction in CENP-C initially leads to an increase in GSC self-renewal, yet this GSC pool is finite and becomes exhausted over time. Through the use of a temperature-sensitive induction of RNAi, it was possible to deplete CAL1 in male GSCs [16]. This led to a loss in CID asymmetry resulting in a symmetric distribution of CID between GSCs and GBs. Furthermore, the number of GSCs per testis was reduced, as well as expression of Stat92E, a key transcription factor for GSC maintenance. Over time, an enlargement of the hub area was observed, an indicator of GSC loss. Taken together, CAL1 depletion in males resulted in failed GSC self-renewal, while CAL1 depletion in females blocked GSCs differentiation or division. By contrast, depletion of CENP-C in females resulted in more GSC self-renewal, eventually leading to a depletion of the GSC pool over time [23]. Ultimately, knockdown of CAL1 or CENP-C in GSCs reduces the stem cell pool in either testes or ovaries [16,23]. Intriguingly, knockdown of the HASPIN kinase also results in more GSC self-renewal in females [17]. It is possible that the targeting of Aurora B kinase to metaphase centromeres via the H3T3P mark could be important for maintaining CID asymmetry.

As an additional means to perturb CID level in GSCs, the over-expression of centromeric proteins was used in females. Indeed, over-expression of CID together with its assembly factor CAL1 shifted the distribution of CID from asymmetric to a symmetric one [17]. A decrease in the ratio of GSCs to CBs was also measured, implying a shift to more stem cell self-renewal. Interestingly, when CAL1 is over-expressed alone (without CID), the GSC/CB balance does not change. Rather the number of both GSCs and CBs increases, suggesting instead that CAL1 promotes proliferation. This result is consistent with CAL1 function in the proliferative capacity of *Drosophila* ISCs and in terminally differentiated EBs [54]. In contrast to CAL1, CENP-C over-expression did not perturb CID asymmetry, nor did it alter the SXL/pMad ratio [23]. Therefore, CENP-C does not appear to be sufficient to drive asymmetry, rather it probably functions in maintaining the correct level of CID asymmetry. It is not yet known whether CID, CAL1 or CENP-C over-expression can perturb the GSC/GB balance in males. Another possibility is that the observed increased number of stem cells relative to daughter cells might be due to dedifferentiation, but this has not yet been investigated.

In summary, over-expression and depletion studies in male and female GSCs support a model in which CID asymmetry at individual pairs of sister centromeres impacts on the number and balance of stem and daughter cells in the niche. However, it is likely that cell fate changes are due to secondary factors such as signalling in the niche or changes at the level of transcription. Whether centromere specification can truly direct stem cell fate, remains enigmatic to test due to difficulties separating this function from its essential role in cell division.

## 7. Future perspectives

Several questions with respect to centromere assembly and asymmetry in male and female GSCs remain unanswered. For instance, how and when is CID asymmetry established? New CID assembly in G2-phase could be an ideal time. The assembly of CID in G2-phase [16,17], before chromosome segregation, might be a unique feature of stem cells. This time frame of CID assembly seems to be conserved in both sexes. Apart from germ cells, G2-phase CID assembly has been observed in *Drosophila* neuronal stem cells [17], but whether this is a common feature for all stem cell types remains unanswered. Otherwise, perhaps it is a biased redistribution of parental CID at the replication fork that establishes asymmetry. DNA and chromatin fibres in *Drosophila* testes unusually show unidirectional fork movement [52], providing a possible mechanism for how CID asymmetry could be established. It will also be important to understand if CID is differentially maintained at stem and daughter cell centromeres. Studies in humans have shown that HJURP is required to maintain CENP-A during DNA replication [73]. It is possible that in *Drosophila* CAL1 and/or CENP-C are required in S-phase to maintain parental CID in GSCs.

Although the asymmetric distribution of centromeric proteins in GSCs is conserved, notable differences in the levels of asymmetry were observed between males and females. In female GSCs, 20% more CID and 30% more CENP-C [17,23] was measured, whereas CID asymmetry in male GSCs was higher, at 50% [16]. The difference in the level of CID asymmetry observed between males and females could be due to experimental quantitation methods. However, it might indicate different underlying mechanisms between sexes. For instance, female and male GSCs systems differ in several aspects, such as a sex-specific JAK-STAT signalling from stem cell niches to regulate GSC self-renewal and maintenance. In female GSCs, JAK-STAT signalling in cap cells triggers the secretion of *Dpp* and *Gbb* ligands, which activates BMP signalling to supress *bam* transcription [33,35,36]*.* In males, it is the secretion of *Upd* ligands from hub cells that activate the JAK-STAT signalling pathway, both in GSCs and CySCs [41,74]. Furthermore in males, *Gbb/Dpp* signalling from both the hub and CySCs activate BMP, leading to *bam* repression in GSCs [44]. Another difference is that each female niche hosts 2 to 3 GSCs, while males are more complex, hosting up to 15 GSCs per niche [4,25,26]. The differential mechanism of spindle orientation in male GSCs [75], which has not been observed in females, might be necessary due to this larger niche.

It is also essential to understand whether properties of centromere asymmetry are stem cell or ACD specific. In male GSCs, sex chromosomes segregate non-randomly while this is not the case for autosomes [76]. If this occurs in female GSCs is not yet known. Investigating this might help to understand better a different—perhaps chromosome-specific—underlying mechanisms in both sexes. Future investigations should also aim to analyse how CENP-A asymmetry and biased sister chromatid segregation affect gene expression. The ability to sort stem and daughter cell populations from tissues, combined with single cell RNA sequencing approaches, will be key. Finally, it will be important to test whether the ability of CENP-A asymmetry to drive cell fate holds true in pluripotent stem cell systems beyond *Drosophila* to elucidate if this phenomenon is conserved in other species.

## Data accessibility

This article has no additional data.
